# Severe Progressive Diffuse Alveolar Hemorrhage in a Patient with Systemic Lupus Erythematosus

**DOI:** 10.1155/2018/9790459

**Published:** 2018-06-07

**Authors:** Munenori Kusunoki, Takeshi Umegaki, Tomohiro Shoji, Kota Nishimoto, Natsuki Anada, Akiko Ando, Takeo Uba, Kanako Oku, Saya Hakata, Satoshi Hagihira, Takahiko Kamibayashi

**Affiliations:** Department of Anesthesiology, Kansai Medical University Hospital, Osaka, Japan

## Abstract

Diffuse alveolar hemorrhage (DAH) refers to the effusion of blood into the alveoli due to damaged pulmonary microvasculature. The ensuing alveolar collapse can lead to severe hypoxemia with poor prognosis. In these cases, it is crucial to provide respiratory care for hypoxemia in addition to treating the underlying disease. Here, we describe our experience with a case involving a 46-year-old woman with severe DAH-induced hypoxemia accompanying systemic lupus erythematosus (SLE). Mechanical ventilation was managed using airway pressure release ventilation (APRV) after intubation. Through APRV-based respiratory care and treatment of the underlying disease, hemoptysis was eliminated and oxygenation improved. The patient did not experience significant barotrauma and was successfully weaned from mechanical ventilation after 25 days in the intensive care unit. This case demonstrates that APRV-based control for respiratory management can inhibit the effusion of blood into the alveoli and achieve mechanical hemostasis, as well as mitigate alveolar collapse. APRV may be a useful method for respiratory care in patients with severe DAH-induced hypoxemia.

## 1. Introduction

Diffuse alveolar hemorrhage (DAH) is a clinicopathological syndrome characterized by intra-alveolar bleeding from damaged pulmonary microvasculature, which can arise due to factors such as pulmonary capillaritis or humoral immune responses [1–3]. This condition can lead to hemoptysis or alveolar collapse resulting in hypoxemia [1–3]. In some cases, DAH can result in acute respiratory failure and death.

DAH is a particularly important potentially fatal complication in patients with systemic lupus erythematosus (SLE) [4], and physicians face difficulties in managing both the underlying disease and respiratory dysfunction in these patients.

This case report describes severe DAH in a 46-year-old woman with SLE who developed severe hypoxemia. Mechanical ventilation was performed using airway pressure release ventilation (APRV). The patient was successfully treated with the combined use of APRV, immunosuppressive therapy, and high-dose corticosteroid therapy.

## 2. Case Presentation

A 46-year-old Japanese woman with known SLE was admitted to our hospital due to sudden weight loss and respiratory distress. Her vitals on arrival were a temperature of 37.1°C, heart rate of 96 bpm, blood pressure of 148/82 mmHg, respiratory rate of 18 per minute, and oxygen saturation of 95% in room air. The patient was given 50 mg of oral prednisolone immediately after admission.

**Figure 1:**
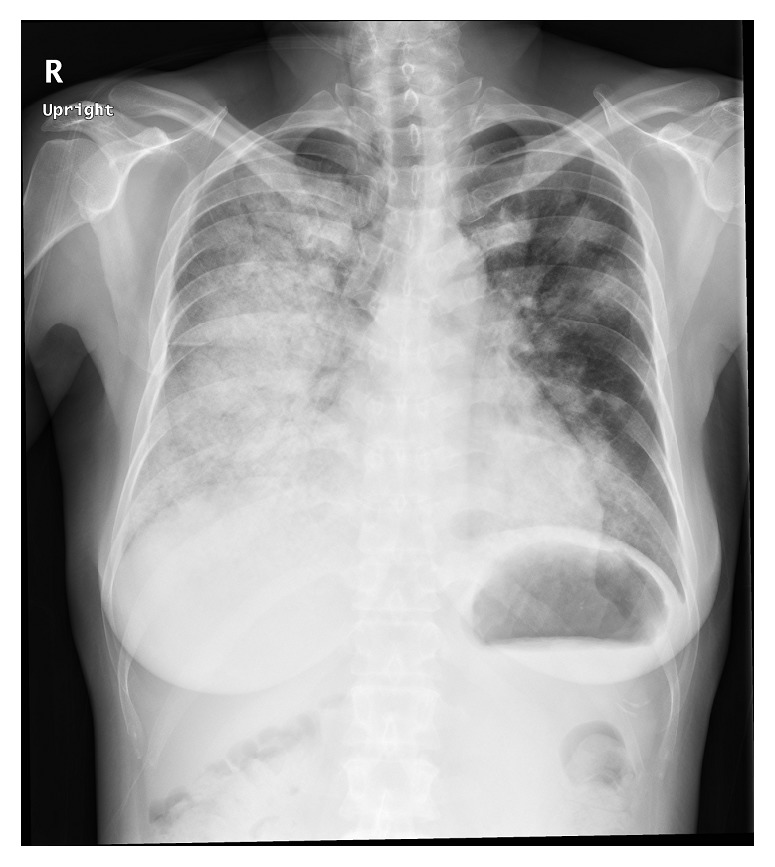
Chest radiograph upon the start of high-dose corticosteroid therapy. Infiltrative shadows are clearly observed in both lung fields.

**Figure 2:**
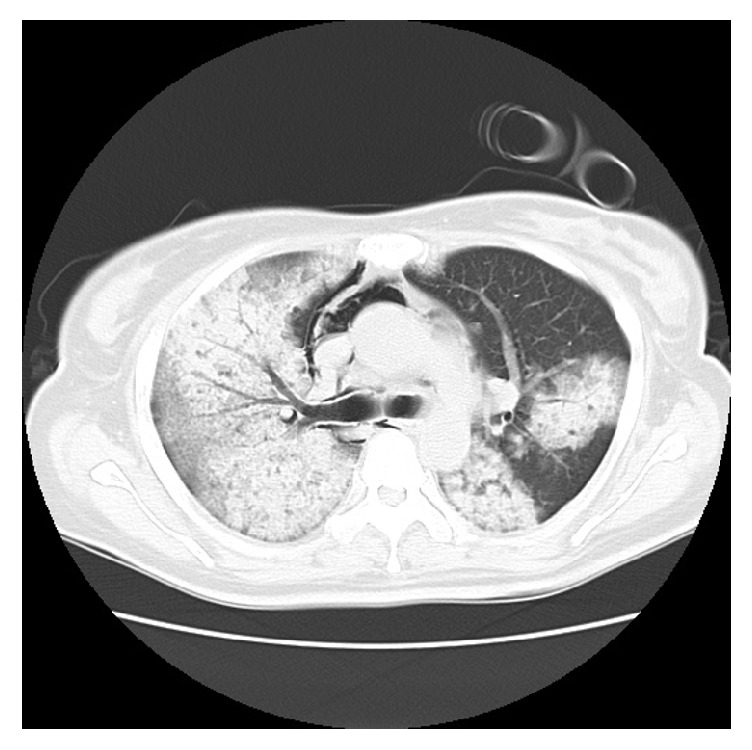
CT scan upon the start of high-dose corticosteroid therapy. The scan shows bilateral pulmonary infiltrates along the peripheral bronchovascular bundles and ground-glass opacities with a panlobular distribution.

Approximately 3 weeks after the start of treatment, the dosage of prednisolone was reduced to 45 mg/day. However, the patient suddenly developed hemoptysis and respiratory distress. A chest X-ray (Figure 1) revealed infiltrative shadows in both lung fields, with greater density in the right lung. A computed tomography (CT) scan showed pulmonary infiltrates along the peripheral bronchovascular bundles and ground-glass opacities (Figure 2). We diagnosed the patient with DAH-induced hypoxemia accompanying SLE and initiated high-dose corticosteroid therapy (methylprednisolone 500 mg/day). However, respiratory distress worsened, and we began immunosuppressive therapy (cyclophosphamide 750 mg/day) 3 days after the start of high-dose corticosteroid therapy. The patient was also transferred to the intensive care unit (ICU) and placed on mechanical ventilation. Although the patient produced copious quantities of bloody secretions after intubation, the secretions were reduced when APRV was set to the highest airway pressure (35 cmH_2_O). We administered oxygen through an oxygen mask (flow rate: 6 L/min) while in the ICU. Under these conditions, the arterial oxygen partial pressure (PaO_2_) was 55.1 mmHg, and the ratio of arterial oxygen partial pressure to fractional inspired oxygen (P/F ratio) was 125.2. APRV resulted in substantial improvements to respiratory function, with a fraction of inspired oxygen (FiO_2_) of 0.60 and a PaO_2_ of 117.6 mmHg.

**Figure 3:**
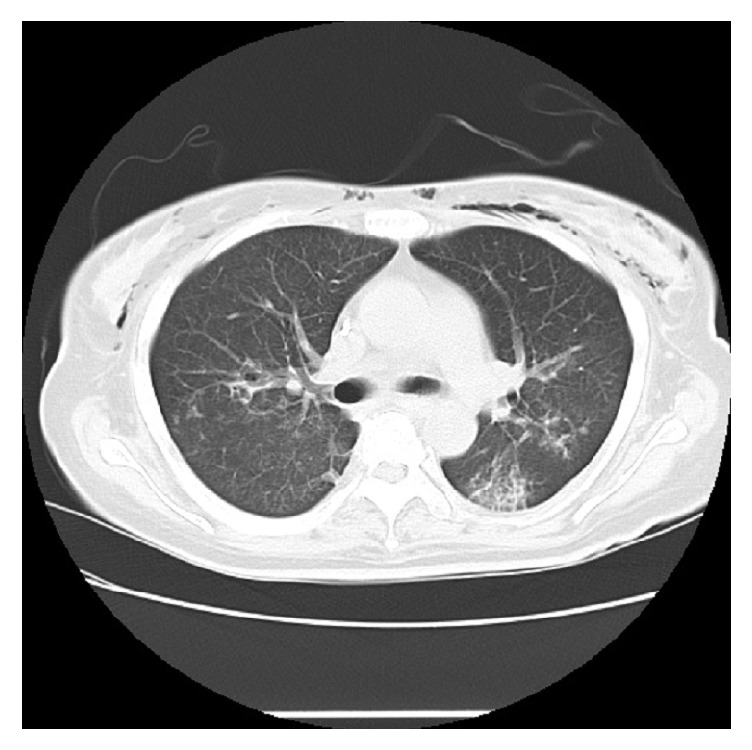
CT scan after extubation. The pulmonary infiltrates and ground-glass opacities had disappeared.

High-dose corticosteroid therapy was administered using 500 mg/day of methylprednisolone for 3 days, 250 mg/day of methylprednisolone for 3 days, and 125 mg/day of methylprednisolone for 3 days, after which the patient was given 50 mg/day of intravenous prednisolone every day while in the ICU. During approximately 3 consecutive weeks of APRV and steroid therapy, the patient demonstrated gradual improvements in respiratory function, and a CT scan after extubation showed that the pulmonary infiltrates and ground-glass opacities had disappeared (Figure 3). Mechanical ventilation was discontinued 25 days after admission to the ICU. The patient was moved to the general ward and continued on oral steroid therapy (methylprednisolone 16 mg/day) until discharge. The patient's respiratory and hemodynamic parameters from admission to ICU discharge are summarized in Table 1. The patient was successfully discharged from hospital 102 days after admission.

## 3. Discussion

The requirement for mechanical ventilation in SLE patients with DAH has been reported to be associated with higher mortality [5, 6]. The case described here provides valuable findings that the use of APRV and high-dose corticosteroid therapy can contribute to the alleviation of respiratory distress in an SLE patient with severe DAH. To the best of our knowledge, this is the first report to describe a case of severe DAH where respiratory dysfunction was successfully resolved with this unique approach.

DAH is associated with primary vasculitis (due to conditions such as microscopic polyarteritis, Wegener's granulomatosis, and Churg-Strauss syndrome) and secondary vasculitis accompanying connective tissue diseases (such as SLE and malignant rheumatoid arthritis) [7]. DAH frequently presents as diffuse bleeding into the alveoli as a result of microvascular injury, and cases like the one described here may result in fatal consequences if treatment is unsuccessful [8, 9].

The development of barotrauma has been reported to have little effect on ventilator parameters such as peak pressure, positive end-expiratory pressure, and tidal volume [10]. However, patients with acute respiratory distress syndrome are at increased risk of barotrauma arising from high airway pressure if consistently exposed to peak pressures of 35 cmH_2_O or more during mechanical ventilation [11]. Therefore, the risk of barotrauma must be considered when performing respiratory management with high peak pressures in these patients.

As there are currently no established evidence-based treatment protocols for DAH, cases are generally treated with high-dose corticosteroid therapy or immunosuppressive therapy (e.g., cyclophosphamide), which target pulmonary capillaritis and vasculitis [12]. However, positive pressure mechanical ventilation can be useful for recruitment maneuvers and hemostasis for collapsed alveoli, such as in the successful treatment of DAH using high-frequency oscillation ventilation [13]. In our patient, the combination of high-dose corticosteroid therapy, immunosuppressive therapy, and APRV was used to improve DAH and acute respiratory distress. APRV is characterized by relatively long high-pressure phases with intermittent short pressure release phases. The short durations for pressure release prevent alveolar collapse and ventilator-associated lung injury and allow for the steady expansion of the alveoli and improvement of oxygenation [14]. The maintenance of positive pressure using APRV in our patient may have contributed to the prevention of intra-alveolar bleeding, improvement of alveolar collapse, and alleviation of respiratory distress.

## 4. Conclusions

This case report describes the successful use of APRV-based respiratory management to inhibit the effusion of blood into the alveoli and achieve mechanical hemostasis, as well as mitigating alveolar collapse. APRV may be a useful method for respiratory care in SLE patients with severe DAH-induced hypoxemia when used in combination with high-dose corticosteroid therapy and immunosuppressive therapy to control the exacerbation of SLE.

## Figures and Tables

**Table 1:** Respiratory and hemodynamic parameters from hospital admission to ICU discharge.

Variables	Oxygen therapy	FiO_2_ (%)	P_high_ (cmH_2_O)	SpO_2_ (%)	PaO_2_ (mmHg)
ICU admission prior to intubation	Mask 6 L/min	0.44	-	≤90	55.1
Two hours after intubation	APRV	0.60	35	100	117.6
ICU Day 7	APRV	0.30	35	100	97.6
ICU Day 14	APRV	0.30	30	100	102.2
ICU Day 21	CPAP	0.30	15	100	100.5
One hour after extubation	HFNC 40 L/min	0.40	-	100	122.9

APRV, airway pressure release ventilation; CPAP, continuous positive airway pressure; FiO2, fraction of inspiratory oxygen; HFNC, high-flow nasal cannula; ICU, intensive care unit; PaO_2_, partial pressure of arterial oxygen; P_high_, peak inspiratory pressure; SpO_2_, oxygen saturation of peripheral artery.
